# IgG4-related kidney disease from the renal pelvis that mimicked urothelial carcinoma: a case report

**DOI:** 10.1186/s12894-015-0041-6

**Published:** 2015-05-27

**Authors:** Hui Zhang, Xinyu Ren, Wen Zhang, Di Yang, Ruie Feng

**Affiliations:** Department of Pathology, Peking Union Medical College Hospital, Chinese Academy of Medical Sciences and Peking Union Medical College, 1 Shuaifu Yuan, Beijing, 100730 PR China; Department of Rheumatology, Peking Union Medical College Hospital, Chinese Academy of Medical Sciences and Peking Union Medical College, Beijing, 100730 PR China

**Keywords:** IgG4-related disease, IgG4-related kidney disease, Renal pelvis, Urothelial carcinoma

## Abstract

**Background:**

IgG4-related kidney disease is a comprehensive term for renal lesions associated with IgG4-related disease, which mainly manifests as plasma cell-rich tubulointerstitial nephritis with increased IgG4+ plasma cells and fibrosis. IgG4-related kidney disease in the renal pelvis is rare.

**Case presentation:**

We describe a 53-year-old Asian woman who was referred to our hospital with a space-occupying renal lesion discovered by medical examination. A physical examination and laboratory evaluation revealed no significant abnormalities. Computed tomography scans showed a soft-tissue mass with an irregular border and mild homogeneous enhancement in the right renal pelvis and calyces. A positron emission tomography/computed tomography scan revealed soft-tissue density shadows with increased radionuclide uptake. To investigate a suspected pelvic carcinoma, a right ureteronephrectomy was performed. A pathologic examination of the renal sections showed a dense lymphoplasmacytic infiltrate rich in IgG4+ plasma cells, with fibrosis beneath the urothelial epithelium of the renal pelvis. Postoperatively, the serum IgG4 level was significantly elevated. The patient was diagnosed with IgG4-related kidney disease.

**Conclusion:**

We present a case of IgG4-related kidney disease mimicking urothelial carcinoma in the renal pelvis. When a buried and solitary hypovascular tumor is detected in the kidney, we must consider IgG4-related kidney disease as a differential diagnosis. Accordingly, elevated serum IgG4, radiologic findings, and pathologic examination may improve the diagnosis.

## Background

IgG4-related disease (IgG4-RD) has recently been proposed and is considered to involve conditions of systemic inflammatory fibrosis, including autoimmune pancreatitis, retroperitoneal fibrosis, chronic sclerosing cholangitis, and inflammatory pseudotumor. IgG4-RD has now been reported in nearly every organ, though it was first identified in the pancreas and salivary glands [1–6]. This disease manifests as organ enlargement or nodular/hyperplastic lesions in various organs concurrently or metachronously as a result of marked infiltration of lymphocytes and IgG4-positive plasma cells as well as fibrosis.

IgG4-related kidney disease (IgG4-RKD) is a comprehensive term for renal lesions associated with IgG4-RD, which mainly manifests as plasma cell-rich tubulointerstitial nephritis (TIN) with increased IgG4+ plasma cells and fibrosis (IgG4-related TIN, IgG4-TIN) [6, 7]. Few data exist on the epidemiological and clinical features of large series of patients. The mean age at diagnosis of the reviewed cases is 65 years, and 73–87 % are men [8]. IgG4-RKD is common and predominantly involves the cortex of the kidney; however, we report a rare case of IgG4-RKD involving a renal pelvic lesion that mimicked urothelial carcinoma.

## Case presentation

A 53-year-old woman was referred to our hospital with a space-occupying renal lesion that was discovered incidentally by an ultrasound scan. The patient’s past medical history was positive for a gastric ulcer.

**Fig. 1:**
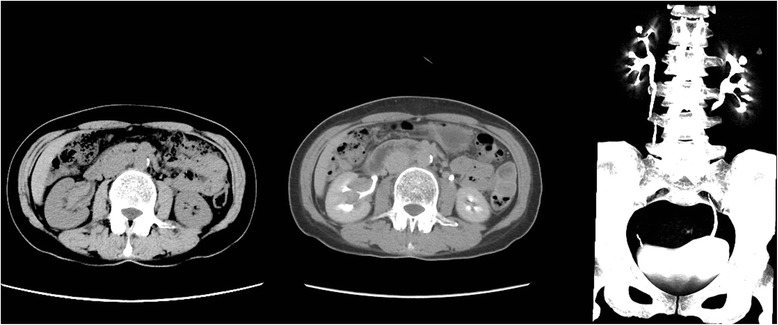
Abdominal computed tomography scans. These scans showed a soft-tissue density mass with an irregular border and mild homogeneous enhancement in the right renal pelvis and calyces

A physical examination revealed no significant abnormalities. Laboratory evaluations, including urinalysis, were within normal limits, and urinary cytology was negative. Computed tomography (CT) scans showed a soft-tissue density mass with an irregular border and mild homogeneous enhancement in the right renal pelvis and calyces (Fig. 1). A positron emission tomography/CT scan revealed soft-tissue density shadows with increased radionuclide uptake, which suggested a malignant lesion.

**Fig. 2:**
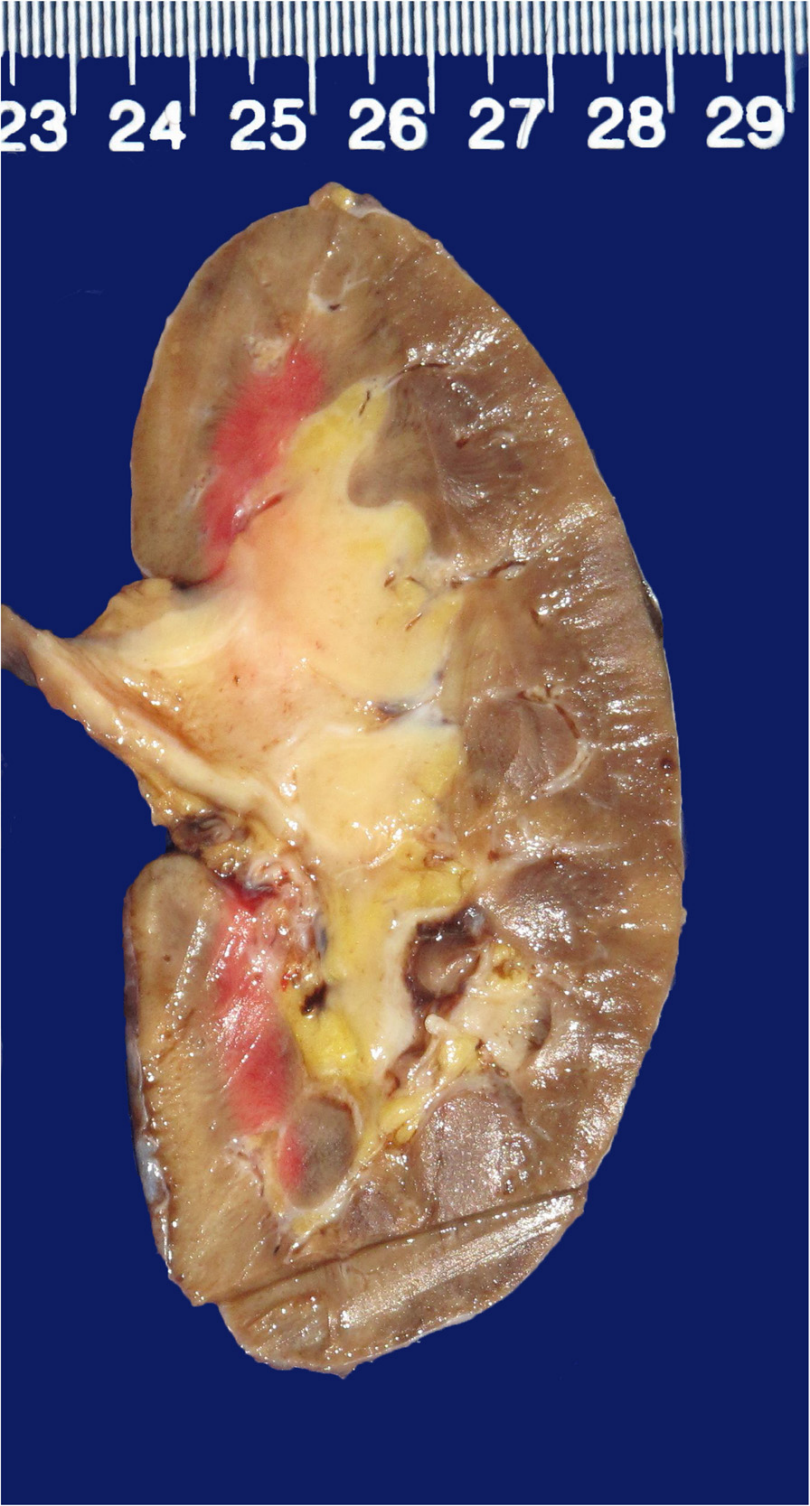
Gross examination revealed thickened mucosa of the renal pelvis

**Fig. 3:**
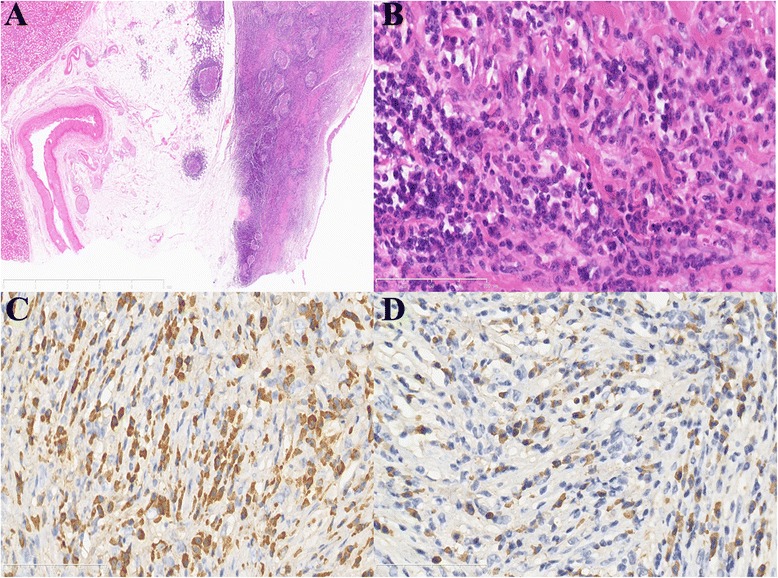
Pathologic examination of the renal sections. (**a**). Numerous lymphoid follicles and prominent fibrosis beneath the urothelial epithelium of the renal pelvis; (**b**). Numerous plasma cells and scattered eosinophils were identified in the interfollicular area. Immunohistochemistry showed numerous IgG+ (**c**) and IgG4+ (**d**) plasma cells

Because pelvic carcinoma was suspected, a right ureteronephrectomy was performed. Gross examination revealed only thickened mucosa of the renal pelvis but no obvious abnormalities in the renal cortex or medulla (Fig. 2). A pathologic examination of the renal sections showed numerous lymphoid follicles and prominent fibrosis beneath the urothelial epithelium of the thickened mucosa (Fig. 3a). Additionally, numerous plasma cells and scattered eosinophils were identified in the interfollicular area (Fig. 3b). The infiltrating lymphocytes and plasma cells did not show significant cytological atypia. Immunohistochemistry demonstrated that more than 40 % of the plasma cells were IgG4+ (Fig. 3c, 3d). All of these histologic changes were confined to the renal pelvis and did not involve the renal parenchyma. The postoperative serum IgG4 level was 3250 mg/L (80–1400 mg/L). The patient was diagnosed with IgG4-RKD and no specific therapy was administered. The postoperative serum IgG4 level decreased from 3250 mg/L to 2450 mg/L at 2 months.

## Discussion

IgG4-RKD is a comprehensive term for renal lesions, including renal parenchymal lesions and renal pelvic lesions, related to IgG4-RD, which is a recently recognized and proposed clinical entity characterized by a dense lymphoplasmacytic infiltrate rich in IgG4+ plasma cells with fibrosis that affects several organs [6, 9]. TIN involving tubules and/or the interstitium of the kidney is the most dominant feature of IgG4-RKD [9]; however, IgG4-RKD in the renal pelvis is rare [7–10]. Here, we report a rare case of IgG4-RKD that mimicked renal pelvic carcinoma.

A comprehensive English and non-English search for all articles pertinent to IgG4-RD of the renal pelvis was conducted using PubMed. Since Naoto Kuroda [11] first reported a case of IgG4-RD arising in the renal pelvis in 2009, six cases of IgG4-RD of the renal pelvis have been reported previously in the world literature (Table 1) [12–16]. The mean age at diagnosis was 59.8 years (range: 49 to 80 years), with a male: female ratio of 1:1. Most patients presented with renal lesions in the left kidney, with a left-to-right presentation ratio of 2:1. Patients visited the hospital with or without complaints of non-characteristic presentations (i.e., flank pain), and none of the patients had hematuria. Hypocomplementemia and elevated serum IgG are characteristic features of IgG4-RD. Elevated serum IgG and IgG4 were found in all patients, but no hypocomplementemia was found in these seven cases, including our case (Table 1). Although hypocomplementemia is a distinct feature of IgG4-RD, a relatively low proportion of patients actually have it.

**Table 1:** Previous reports of IgG4-related kidney disease arising in the renal pelvis

Ref.	Age (years)	Sex	Manifestations	Hypocomplementemia	Extra-renal lesions	Location	Serum IgG4	Therapy	Findings at follow-up
12	58	M	None	NA	IgG4-related MD	Left	Elevated	Surgery + corticosteroid	Dead
13	80	M	NA	NA	None	Right	NA	Surgery	NA
14	69	M	NA	NA	None	Right	Elevated	Surgery + corticosteroid	Regression of the manifestations
11	49	F	None	None	IgG4-related MD	Left	Elevated	Surgery	NA
15	49	F	Bilateral lumbago	NA	None	Left	Elevated	Corticosteroid	Regression of the manifestations
16	54	F	Left flank discomfort and palpebral edema	None	None	Left	Elevated	Corticosteroid	Regression of the manifestations

F, female; M, male; MD, mikulicz’s disease; NA, unavailable

Patients with IgG4-RD often have lesions in several organs, either synchronously or metachronously, although others may show the involvement of only a single organ. Renal lesions are recognized as extra-pancreatic manifestations of IgG4-RD; the condition can develop as IgG4-RKD singly or associated with the lesions of other organs. In previous cases, renal and extra-renal (salivary gland) involvements in IgG4-RD have presented simultaneously in two patients [11, 12]. In the current case, a systemic examination showed no other abnormal findings, inclusive of the salivary glands, lacrimal glands, and pancreas. Thus, the condition was diagnosed as IgG4-RD isolated in the renal pelvis without the involvement of other organs.

On the basis of the results of a diagnostic algorithm procedure and with references to several diagnostic criteria for AIP, Mitsuhiro Kawano [6] proposed diagnostic criteria for IgG4-RKD: (1) presence of some kidney damage, as manifested by laboratory examination; (2) kidney imaging studies showing abnormal renal findings, i.e., multiple low-density lesions on enhanced CT; (3) elevated serum IgG4 levels exceeding 135 mg/dL; (4) renal histology showing dense lymphoplasmacytic infiltration with infiltrating IgG4+ plasma cells and fibrosis; and (5) extra-renal histology showing prominent lymphoplasmacytic infiltration with infiltrating IgG4+ plasma cells. The diagnosis is classified into three stages—definite, probable, and possible—according to the combinations of the above conditions. In their diagnostic criteria, abnormal renal imaging findings were essential for making a definitive diagnosis. In the present case, all of these conditions, including imaging studies that identified low-density lesions, pathologic examinations that revealed characteristic changes, and elevated serum IgG4, prompted the definitive diagnosis of IgG4-RKD.

A rapid response to corticosteroid therapy is a characteristic feature of IgG4-RD, and corticosteroids are typically the first line of therapy, although no controlled trial has been performed. Moreover, the protocol used for corticosteroid therapy varies among countries and institutions [1]. Because of the decreased level of serum IgG4 after ureteronephrectomy, our patient received a close follow-up without corticosteroid therapy. In the reported cases, patients with IgG4-RKD arising from the renal pelvis were treated according to different strategies, including surgical treatment alone for two patients, corticosteroid therapy alone for two patients, and surgical and corticosteroid treatment for the remaining two patients. The renal lesions improved or resolved after the corticosteroid treatment in three patients who received corticosteroid treatment (Table 1). Takahashi and colleagues [17] also found that lesions progressed in three IgG4-TIN patients receiving no corticosteroid treatment or surgical resection and that lesions regressed in all IgG4-TIN patients who underwent corticosteroid treatment. These observations indicate that effective interventions should begin as soon as possible for irreversible fibrosis in IgG4-RKD. Because corticosteroid treatment has a remarkable effect in this type of disease, at least in the short term, this treatment is vital to avoid unnecessary surgery. CT-guided biopsy or laparoscopic biopsy of the original tumor might help to rule out malignancy.

Recent studies have revealed several characteristic clinical features of IgG4-RKD, including predominance in middle-aged to older men, frequent association with IgG4-RD in other organs, high levels of serum IgG and IgG4, and a good initial response to corticosteroids. However, longer follow-up data for IgG4-RKD, including relapse information, are still sparse. Takako Saeki and his colleagues [18] retrospectively analyzed the longer-term clinical course of 43 patients with IgG4-TIN in detail in a larger cohort. This analysis included the largest series on the long-term outcomes of corticosteroid treatment of IgG4-RKD. Saeki et al. showed that 1 month after the start of treatment, most of the abnormal serology and radiology parameters had improved, and relapse of IgG4-related lesions occurred in 8 of 40 treated patients. These studies indicate that the response of IgG4-RKD to corticosteroids is rapid and partial, and that irreversible lesions may remain, especially in patients with advanced renal damage. Patients with renal dysfunction should receive corticosteroid therapy, although spontaneous improvement of lesions can also occur in IgG4-TIN and the indications for corticosteroid therapy in IgG4-RKD have not been established. Careful attention should be paid to renal function during follow-up without therapy [18, 19]. A large-scale prospective study is necessary to determine a more useful treatment strategy for IgG4-RKD.

## Conclusions

We present a case of IgG4-RKD that mimicked urothelial carcinoma in the renal pelvis. When a deep and solitary hypovascular tumor is detected in the kidney, we must consider IgG4-RKD as a differential diagnosis. Accordingly, elevated serum IgG4, radiologic findings, and pathologic examination may be helpful for the diagnosis.

### Consent

Written informed consent was obtained from the patient for publication of this case report and any accompanying images.

## Abbreviations

IgG-RD: IgG-related disease
IgG-RKD: IgG-related kidney disease
TIN: Tubulointerstitial nephritis
CT: Computed tomography
